# The Sale of Poisons

**Published:** 1858-03

**Authors:** 


*The sale of Poisons.*—We extract the following from the Boston Medical Journal, and hope indeed that it may have some effect in remedying this great evil, under which we now labor. It is now as easy for a person to go into a drug store and buy a dime's worth of laudanum as it is to buy a loaf of bread. The small profits which druggists obtain from such persons, should not be sufficient to make them forget that when they sell poison without the order of a physician, they are violating the moral law, if not the law of the land. It seems to us, that if instead of making an impossible law against the vending of intoxicating beverages, the legislature had taken up the subject of the unauthorized selling of poisons, it would have provoked less ridicule and produced more benefit: F.

We have more than once called attention to the importance of a law regulating the sale of poisons to irresponsible persons, and we hope that the present legislature may deem it worth while at least to inquire into the expediency of some such measure, with a view of preventing suicide and murder, which crimes are really now committed with ease, since any person may procure laudanum, arsenic, or any other active poison, simply by paying for it. The subject was again forced on our notice a few days since, by a case in which a young woman nearly accomplished her purpose of self-destruction, through the inconsiderateness of an apothecary, who sold her half an ounce of laudanum, without the slightest hesitation. Fortunately the patient had taken a full meal before swallowing the laudanum, and as medical assistance was at once obtained, her life was saved, though with difficulty, as it was only by threats that she could be induced to take the repeated emetics which were necessary to evacuate the stomach.

We need a law which renders the purchase of active poisons for criminal purposes more difficult than it now is. They ought never to be sold at retail without a prescription from a physician. At least they ought never to be sold to a person applying alone: he should be required to be accompanied by a friend knowing the nature and effects of the article purchased, and a registry should be kept containing the names of the purchaser and witness, the date, the name and quantity of the drug sold, with the alleged purpose for which it is to be used.

It is one of the inconveniences of our form of government that it is difficult to enact laws for the preservation of human life, or to execute them when enacted. It is thought that we have a right to commit murder or suicide, provided we choose to abide by the consequences; to prevent the sale of poisons or other means of destruction is considered as infringing on the liberty of the subject. In older countries the case is different; in France or England poison cannot be bought at retail except under restrictions similar to those we have named above. But while we are waiting till experience shall make us wiser, would it not be worth while for the apothecaries to take the matter into their own hands, and refuse to sell active medicines without a physician's prescription, or at least without requiring the presence of a witness to the sale? We do not see how they could lose anything by adopting this precaution, except the few cents they would gladly forfeit to prevent a crime; on the contrary, they would gain the esteem of the community, and would, we have no doubt, find an increased amount of patronage. We believe that these means, or similar ones, are adopted by some of our best apothecaries; humanity demands that they should be adopted by all.

